# Supplementary material for: Development of Livestock-Associated Methicillin-Resistant Staphylococcus aureus (LA-MRSA) Loads in Pigs and Pig Stables During the Fattening Period
Source: Vet Sci. 2024 Nov 11;11(11):558. doi: 10.3390/vetsci11110558 (PMC11599088; doi:10.3390/vetsci11110558)
Supplement: Supplementary file 1 [file vetsci-11-00558-s001.zip › vetsci-3218056-supplementary.pdf]

Supplementary Table S1

Table S1. Sampling from seven batches of 500 pigs (nasal swabs, air and dust).

	Week no.																																		
Batch	39	40	41	42	43	44	45	46	47	48	49	50	51	52	1	2	3	4	5	6	7	8	9	10	11	12	13	14	15	16	17	18	19	20	21
1	X			X				X																											
2		X			X				X																										
3			X			X				X																									
4													X			X				X															
5															X			X				X													
6																X			X				X												
7																												X			X				X

X means that sampling was performed that week. Every batch was sampled three times over an eight week period.
